# A Single-Center Study of the Impact of the COVID-19 Pandemic on the Organization of Healthcare Service Delivery to Patients with Head and Neck Cancer

**DOI:** 10.3390/cancers15194700

**Published:** 2023-09-24

**Authors:** Magdalena Kanicka, Mariusz Chabowski, Monika Rutkowska

**Affiliations:** 1 Oncological Package Unit, 4th Military Teaching Hospital, 50-981 Wroclaw, Poland; kanicka.magda@gmail.com; 2 Department of Surgery, 4th Military Teaching Hospital, 50-981 Wroclaw, Poland; 3 Department of Nursing and Obstetrics, Division of Anesthesiological and Surgical Nursing, Faculty of Health Science, Wroclaw Medical University, 50-367 Wroclaw, Poland; 4 Department of Otolaryngology, Head and Neck Surgery, 4th Military Teaching Hospital, 50-981 Wroclaw, Poland; chirurgiamrutkowska@gmail.com

**Keywords:** COVID-19, DILO (diagnosis and oncological treatment), head and neck cancer, pandemic, delays in healthcare delivery

## Abstract

**Simple Summary:**

The aim of this study was to assess the influence of the COVID-19 pandemic on the diagnosis and treatment of head and neck cancer (HNC) patients between 2018 and 2022. We analysed the medical records of 625 patients. During the COVID-19 pandemic, compared with the pre-COVID-19 pandemic period, the duration of the cancer diagnostic process was significantly longer, the proportion of patients admitted on the basis of a DILO card issued by a primary care physician was significantly higher, and the majority of cancer patients with a planned oncological treatment lived in urban areas.

**Abstract:**

The aim of this study was to identify and assess the impact of the COVID-19 pandemic on the diagnosis and treatment of head and neck cancer (HNC) patients of the Department of Otolaryngology, Head and Neck Surgery of the 4th Military Teaching Hospital in Wroclaw for whom oncological treatment was planned by a cancer case board between March 2018 and February 2022. We analysed the medical records of 625 patients. In order to verify whether the relationships between the analysed features were statistically significant, the chi-square test of independence and the Student’s *t*-test for independent samples were used (*p* < 0.05). Our analysis showed that the impact of the pandemic on the organization of health service delivery to HNC patients was not uniform. The largest difference in the number of formulated treatment plans was observed at the beginning of the pandemic (22.1% reduction compared with the year before the pandemic). During the pandemic, the proportion of patients admitted on the basis of a DILO (diagnosis and oncological treatment) card issued by a primary care physician, instead of a regular referral to hospital, issued also by a primary care physician, was significantly higher compared with the that during the pre-pandemic period. The majority of cancer patients with a oncological treatment planned during the pandemic lived in urban areas. During the pandemic, the number of patients with more-advanced-stage cancer, assessed on the basis of the type of planned treatment (radical vs. palliative), did not increase compared with that during the pre-pandemic period. However, our follow-up period was quite short. It is necessary to intensify activities aimed at promoting health and increasing health awareness in people living in rural areas and setting long-term priorities and objectives for health policies at the national, regional and local levels, with particular focus on this group of people.

## 1. Introduction

Head and neck cancer (HNC) is a significant clinical and social problem. In recent years, it has accounted for 5.5–6.2% of all malignancies in Poland, which translates to approximately 5500–6000 new HNC cases each year. Similar incidence rates were reported for other European countries and the USA [1,2,3,4,5,6]. In order to improve and accelerate the diagnosis and treatment of patients with suspected or confirmed malignant cancer, a fast-track cancer pathway was introduced in Poland. In order to be eligible for the fast-track scheme (treatment), patients need to have a DILO (cancer diagnosis and treatment) card [7]. The card is issued by primary care physicians or specialists when they suspect or have diagnosed cancer in their patient. The aim is to make a final diagnosis within a maximum of 7 weeks from the date at which the patient was placed on a waiting list for consultation with a specialist [7].

In March 2020, Poland reported its first case of SARS-CoV-2 and introduced mitigation measures based on traditional anti-epidemic strategies, such as movement and social gathering restrictions, social distancing measures, self-isolation and quarantine. Healthcare institutions were adapted to manage a large number of patients with severe COVID-19 infection. This led to changes in the organization of cancer care, such as the provision of teleconsultation services; reduction in the number of diagnostic tests performed; and suspension of planned, less-urgent procedures. The national authorities decided to limit non-emergency health services, suspending emergency care systems in numerous hospitals [8]. Consequently, the health of patients suffering from acute conditions was put at risk. Cancer patients requiring immediate access to healthcare services were significantly affected by the COVID-19 pandemic, too. At the beginning of the COVID-19 pandemic, some healthcare providers advocated delays in delivering care to patients with mild symptoms or less aggressive HNC forms. This approach was motivated by the fact that these patients were vulnerable to pulmonary complications associated with the virus. That recommendation seemed to prioritize patient safety. However, delays in providing care and performing surgeries in HNC patients were observed. This, in turn, had a negative impact on tumour burden and overall survival [8,9]. The COVID-19 pandemic and its associated restrictions ended in March 2022.

The Aim of this Study

The aim of this study was to identify and assess the impact of the COVID-19 pandemic on the diagnosis and treatment of head and neck cancer patients of the Department of Otolaryngology, Head and Neck Surgery of the 4th Military Teaching Hospital in Wroclaw for whom cancer treatment was planned by a cancer case board.

## 2. Material and Methods

We analysed the medical records of 625 HNC patients of the Department of Otolaryngology, Head and Neck Surgery of the 4th Military Teaching Hospital in Wroclaw for whom cancer treatment was planned by a cancer case board between 1 March 2018 and 28 February 2022. All the patients were included in a further analysis. Both the patients’ sociodemographic data, i.e., gender, place of living, etc., and clinical variables, i.e., diagnosis, type of cancer, type of treatment, etc., were collected.

### 2.1. Inclusion Criteria

The inclusion criteria were patients with a first diagnosis of a head and neck solid neoplasm in the nose and paranasal sinuses, nasopharynx, oral cavity, oropharynx, larynx, hypopharynx, or salivary glands or those with a first diagnosis of neck lymph node metastases from cancer of unknown primary (CUP); inverted papillomas of the sinus and tumours of the parotid gland (Whartin’s tumour and mixed tumour); or recurrent tumours, which are defined as cancer at a postoperative site. The ICD-10 codes are C00, C02, C03, C04, C05, C06, C07, C08.0, C08.1, C09, C10, C11, C30, C31, C32, C69.9, C76.0, C80, D11.0 and D14.0.

### 2.2. Exclusion Criteria

The exclusion criteria were patients in follow-up for HNC; patients with relapsing HNC; patients with thyroid neoplasms; patients with head and neck haematological neoplasms; and patients with head and neck cutaneous neoplasms.

The patients were divided into two groups: those accessing services before the COVID-19 pandemic (between 1 March 2018 and 28 February 2020) (pre-COVID-19 pandemic group) and those accessing services during the pandemic (between 1 March 2020 and 28 February 2022) (COVID-19 pandemic group).

### 2.3. Bioethics Committee

This study was approved by the Bioethics Committee of the Military Chamber of Physicians and Dentists in Warsaw (No 41/2023).

### 2.4. Statistical Analysis

Statistical analysis was performed using Microsoft Office Excel 2016. In order to verify whether the relationships between the analysed features were statistically significant, we used the chi-square test of independence and the Student’s *t*-test for independent samples (level of significance: *p* < 0.05).

## 3. Results

Our data analysis showed that, in the analysed period, cancer treatment plans were formulated for 625 patients: 50.4% (*n* = 315) of the patients received treatment plans before the COVID-19 pandemic and 49.6% (*n* = 310) received treatment plans during the COVID-19 pandemic. The Department of Otolaryngology, Head and Neck Surgery of the 4th Military Teaching Hospital in Wroclaw is one of the two recommended head and neck cancer centres in the Lower Silesia Province.

The mean age of the patients in the pre-COVID-19 pandemic group and the COVID-19 pandemic group was 62 years (median—62 years) and 63 years (median—65 years), respectively. Both before and during the pandemic, a larger percentage were men, i.e., 63.5% men vs. 36.5% women and 59.0% men vs. 36.5% women, respectively.

**Figure 1:**
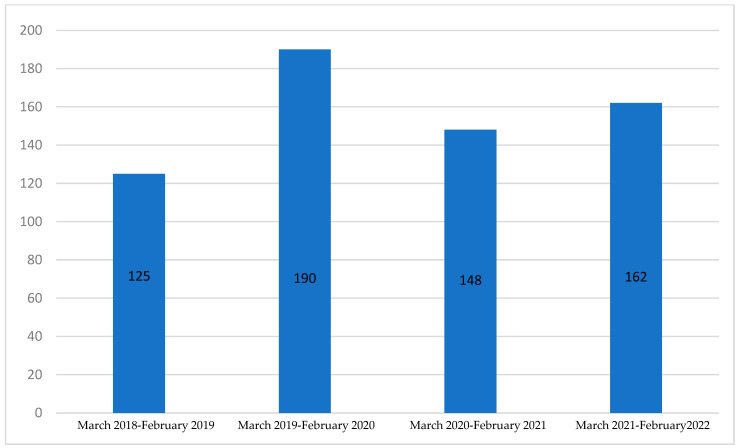
Number of patients for whom cancer treatment plans were formulated by a cancer case board in consecutive years starting from 1 March 2018 to 28 February 2022.

The number of cancer treatment plans formulated by a cancer case board in the first year of our analysis, between 1 March 2018 and 28 February 2019, was 125. In the following year, the number was higher by 52.0% and amounted to 190 (Figure 1). In the first year of the COVID-19 pandemic (1 March 2020–28 February 2021), the number of cancer treatment plans formulated by a cancer case board was lower by 22.1% compared with the year before the COVID-19 pandemic and amounted to 148. In the following year (1 March 2021–28 February 2022), the number amounted to 162 and was higher by 9.5% compared with the year before and lower by 14.7% compared with the last year before the COVID-19 pandemic.

The patients of the Department of Otolaryngology, Head and Neck Surgery of the 4th Military Teaching Hospital in Wroclaw included in the study had HNC at the following sites: oral cavity, pharynx, lip, paranasal sinuses, salivary glands and larynx. A detailed division of the patients according to diagnosis is shown in Table 1. In 3 out of 20 analysed diagnoses, statistically significant differences were found with regard to the distribution of patients. During the pandemic, the patients with tongue cancer (ICD-10: C02) constituted 3.9%, while in the pre-pandemic period, this percentage was only 0.6% (*p* = 0.006). With regard to neck cancer (ICD10–C76.0), the percentage of patients was 1.6% in the period before the pandemic, while there was no such case during the pandemic (*p* = 0.026). The third difference (*p* = 0.012) in the distribution of diagnoses was found with regard to lip cancer (ICD-10 C00). A significantly higher percentage of patients with this diagnosis (2.9%) was noted in the pre-pandemic period than during the pandemic (0.3%). With regard to the rest of the 17 diagnoses, the percentage distribution of patients was similar.

Of the patients in the pre-COVID-19 pandemic group, 70.8% had malignant tumours and 29.2% had benign tumours (inverted papilloma and mixed tumour). Of the patients who received cancer treatment plans between 1 March 2020 and 28 February 2022, 69.4% had malignant tumours and 30.6% had benign tumours (*p* = 0.695) (Table 2); 99.4% of patients in the pre-COVID-19 pandemic group and 98.1% of patients in the COVID-19 pandemic group had primary cancer (*p* = 0.148); and 3.8% of patients in the pre-pandemic group and 4.5% of patients in the COVID-19 pandemic group had a second cancer at another site (*p* = 0.658). Of the patients in the pre-COVID-19 pandemic group, 36.5% were women and 63.5% were men, whereas of the patients in the COVID-19 pandemic group, 41.0% were women and 59.0% were men (*p* = 0.252). Our study included patients living in urban and rural areas. The proportion of residents of urban areas was significantly higher among patients in the COVID-19 pandemic group compared with patients in the pre-COVID-19 pandemic group (79.0% vs. 62.5%) (*p* < 0.001). Of the patients, 84.2% received radical treatment and 15.8% received only palliative treatment. There were no significant differences (*p* = 0.283) in the number of patients for whom radical vs. palliative treatment was planned between the pre-COVID-19 pandemic and the COVID-19 pandemic period. The patients were admitted on the basis of a regular referral or a DILO card issued by a primary care physician. The percentage of patients admitted on the basis of a DILO card was significantly higher during the COVID-19 pandemic period (23.5%) compared with during the pre-COVID-19 pandemic period (16.8%) (*p* = 0.036).

We also analysed the mean duration of the cancer diagnostic process. The oncological diagnostics were defined as a period from the day of the first visit to the specialist to the day of the last visit, when the physician consulted all test results, i.e., histopathological examination confirming the diagnosis of cancer and imaging tests enabling determination of TNM stage, and finally referred the patient for the cancer case board. In the period from 1 March 2018 to 28 February 2020, the mean duration of the diagnostic process was 19 days (median—16 days). In the period from 1 March 2020 to 28 February 2022, the mean duration of the diagnostic process was 23 days (median—20 days) (*p* = 0.008).

We selected 438 patients with the confirmed malignant head and neck tumours out of the 625 patients enrolled initially into the study. We analysed those patients once again, taking into account the features discussed above (Table 3).

In the group of patients in the pre-COVID-19 pandemic with malignant head and neck tumours, 30.9% were women and 69.1% were men, whereas in the COVID-19 pandemic, 31.6% were women and 68.4% were men (*p* = 0.252). The proportion of residents of the urban areas was significantly higher among patients in the COVID-19 pandemic group compared with that for patients in the pre-COVID-19 pandemic group (77.7% vs. 66.4%) (*p* = 0.008). We also confirmed significant differences between the pre-COVID-19 pandemic and the COVID-19 pandemic period in the number of patients with malignant head and neck tumours with respect to the type of admission (82.5% vs. 71.6%) (*p* = 0.007). But, there were no significant differences in the number of patients for whom radical vs. palliative treatment was administered between the pre-COVID-19 pandemic and the COVID-19 pandemic period (79.8% vs. 74.9%) (*p* = 0.217).

## 4. Discussion

In the present study, we analysed the impact of the COVID-19 pandemic on HNC patients over the course of a full 24-month period. The available analyses of the impact of the pandemic on the treatment of cancer patients have focused on the initial period of the pandemic. There are no analyses that cover the entire pandemic period.

Our analysis showed that the impact of the pandemic on the organization of healthcare service delivery to the HNC patients of the Department of Otolaryngology, Head and Neck Surgery of the 4th Military Teaching Hospital in Wroclaw was not uniform. The largest difference in the number of treatment plans formulated by a cancer case board was observed at the beginning of the pandemic (21.1% reduction compared with the year before the pandemic). This is consistent with the data published in a report by the Maria Sklodowska-Curie National Research Institute of Oncology, stating that the number of new cancer diagnoses in Poland in 2020 was lower by approximately 20% compared with 2019 [8]. A similar reduction in new cancer diagnoses was observed in other countries [10,11,12,13,14,15,16]. This may have been due to decisions made by national authorities or particular facilities as well as decisions of individual patients, who, for many months, especially in the initial period of the pandemic, avoided visiting healthcare settings despite clear, worrying symptoms. However, it should be noted that we found no difference in the number of patients provided with cancer healthcare between the two-year period of the pandemic and the two-year period before the pandemic. This may be explained by the fact that patients who initially were afraid of catching the virus and put off getting themselves diagnosed and treated later decided to seek medical help, which made it possible to compensate for the losses associated with the initial period of the pandemic. Moreover, cancer centres remained open throughout the pandemic, whereas other hospitals were mainly responsible for treating patients with COVID-19. This phenomenon was described in a study by Szewczyk et al. [17], which included HNC patients treated at the Cancer Centre in Poznan. The authors compared the characteristics of patients diagnosed with HNC during the 12-month pre-pandemic period prior to the implementation of pandemic-related restrictions in Poland in March 2020 with those of patients diagnosed and treated during the pandemic (March 2020–February 2021). The authors noted that the number of patients who presented to the Multidisciplinary Tumour Board increased by 22% from the pre-pandemic period to the pandemic period. Similar findings were reported for cancer centres by other authors [18,19].

In our study, we found no increase in the number of patients with more-advanced-stage cancer, on the basis of the type of treatment planned (radical vs. palliative) between the pre-pandemic and the pandemic period. However, our follow-up period was quite short. Similarly, a study by Szewczyk et al. [17] found no significant differences between the pre-pandemic period and the pandemic period in the overall percentage of locally advanced cases (stages T3–T4) and in the proportions of patients with particular tumour sites. A study by Balk et al. [20] including patients with newly diagnosed or recurrent head and neck squamous cell carcinoma (HNSCC) found no significant differences with regard to the T stage, N stage and UICC stage between the pre-pandemic period and the pandemic period. A study by Solis et al. [9] based on a retrospective review of patients with newly diagnosed HNSCC found that the proportion of tumours classified as T3/T4 in the pandemic period was higher compared with that during the pre-pandemic period and that the median tumour size was larger during the pandemic period. Similarly, Kiong et al. [21] found that the median primary tumour size was significantly larger and the T stage was more advanced for mucosal subsites in HNC patients presenting during the COVID-19 pandemic. Ralli et al. [22] from the Sapienza University of Rome found that during the pandemic period, the number of cancer patients undergoing surgery was lower by 12.90%, whereas the number of patients treated exclusively with non-surgical approaches was higher by 18.42% compared with the period from 10 March 2019 to 9 March 2020.

Due to the introduced restrictions, personal contact with primary care physicians during the pandemic was limited both in the city and in the countryside. However, in accordance with the guidelines on the prevention and counteracting of COVID-19, the Minister of Health enabled the provision of healthcare services in the form of teleconsultations. The sudden development of telemedicine, which became a necessity, made it possible to maintain contact with the patient and to monitor the treatment process. Poland-wide data showed that the number of DILO cards issued in 2020 decreased compared with that in 2019 but remained at a similar level to that in 2018. The largest percentage decrease concerned cards issued by primary care physicians [8]. In the present study, we assessed changes in the number of patients admitted on the basis of a DILO card and in the number of patients admitted on the basis of a regular referral. During the COVID-19 pandemic, the proportion of patients presenting to our department with a DILO card issued by a primary care physician (instead of a regular referral) was significantly higher compared with that during the pre-pandemic period. This may have been due to the fact that primary care physicians preferred to issue DILO cards (instead of referrals to the outpatient clinics) for their patients in order to put them on the fast path to an appointment with a specialist and to shorten the waiting time for the first consultation. In our opinion, the decrease in the number of patients who consulted doctors from rural areas during the pandemic may have resulted from the patients’ fear of healthcare facilities and possible COVID infection.

The available medical record data did not allow us to clearly determine the time between the onset of the first symptoms and the patient’s attempt to contact a physician. The DILO system only allows for tracking the patient’s history from the moment their doctor first suspects cancer to the onset of treatment. However, it is during this initial period that the longest delays occur. Our study showed that the pandemic significantly affected the duration of the cancer diagnostic process, which increased by a mean of 4 days compared with that during the pre-pandemic period. The literature in this area provides various results. In a study by Szewczyk et al. [17], the mean time from the first visit to the multidisciplinary tumour board meeting differed slightly (but not significantly) between the pre-COVID-19 pandemic period and the period of the COVID-19 pandemic. Zubair [23] confirmed that there was no delay in the initiation of first treatment after the decision to treat. In contrast, Kourtidis et al. [24] found that the symptom-to-diagnosis interval was longer during the COVID-19 pandemic and that the interval from diagnosis to treatment and the interval from treatment initiation to the end of treatment during the pandemic period were approximately the same as in the pre-pandemic period. Psycharis [25] noticed that pandemic patients experienced a significant time reduction compared with pre-COVID pandemic patients with regard to the date first seen by a HNC service until the start of treatment and the date first seen by a HNC service until first presentation at the tumour board. According to the aforementioned report by the National Research Institute of Oncology, fewer non-cancer patients attended their planned imaging appointments during the pandemic, which sped up the diagnostic process for cancer patients [8].

One of the social factors that have an impact on health is the place of residence [2,26,27,28,29]. People living in rural areas may have poorer access to healthcare services, education and other resources that may have a positive impact on health [30]. Thus, living in a rural area may be a factor that negatively affects health. Our study showed that most of the patients for whom treatment plans were formulated during the COVID-19 pandemic lived in urban areas. Thus, the COVID-19 pandemic exacerbated these health inequalities [8]. Access to primary care physicians during the pandemic has been limited both in cities and rural areas. However, the sudden development of telemedicine made it possible to maintain contact with the patient and to monitor the treatment process. In our opinion, the decrease in the number of patients from rural areas may have resulted from the patients’ fear of the COVID infection. Therefore, it is necessary to intensify activities aimed at promoting health and increasing health awareness in people living in rural areas and to set long-term priorities and objectives for health policies at the national, regional and local levels, with particular focus on this group of people.

### Limitation of Study

The patients included into the study were not analysed according to their stage of disease. In our paper, we focused on the impact of the pandemic on the diagnostic and therapeutic process of patients diagnosed with head and neck cancer, along with the factors influencing this process. We analysed neither the stage of the disease nor the precise localization of the cancer.

## 5. Conclusions

The impact of the COVID-19 pandemic on the organization of healthcare service delivery to HNC patients at the 4th Military Teaching Hospital in Wroclaw was not uniform. The largest difference in the number of treatment plans formulated by a cancer case board was observed at the beginning of the COVID-19 pandemic.

During the COVID-19 pandemic, compared with the pre-COVID-19 pandemic period, the following was observed:

- The duration of the cancer diagnostic process was significantly longer;
- The proportion of patients admitted on the basis of a DILO card issued by a primary care physician, instead of a regular referral, also issued by a primary care physician, was significantly higher;
- The majority of cancer patients with a oncological treatment planned during the COVID-19 pandemic lived in urban areas;
- The number of patients with more-advanced-stage cancer, assessed on the basis of the type of planned treatment (radical vs. palliative), did not increase.

It is necessary to intensify activities aimed at promoting health and increasing health awareness in people living in rural areas and setting long-term priorities and objectives for health policies at the national, regional and local levels, with particular focus on this group of people.

## Figures and Tables

**Table 1:** Number of patients with confirmed benign or malignant head and neck tumours by tumour site before and during the COVID-19 pandemic.

	Period	Pre-COVID-19 Pandemic		COVID-19 Pandemic		Total		*p*-Value- Chi^2^ Test
Tumour Site		*n*	%	*n*	%	*n*	%	
Bottom of the oral cavity		21	6.7	17	5.5	38	6.1	0.536
Gum		21	6.7	16	5.2	37	5.9	0.425
Pharynx		6	1.9	6	1.9	12	1.9	0.978
Nasal cavity		6	1.9	3	1.0	9	1.4	0.326
Tongue		44	14.0	39	12.6	83	13.3	0.609
Larynx		62	19.7	60	19.4	122	19.5	0.918
Tonsil		2	0.6	12	3.9	14	2.2	0.006
Nose		1	0.3	2	0.6	3	0.5	0.553
Eye socket		4	1.3	4	1.3	8	1.3	0.982
Palate		10	3.2	18	5.8	28	4.5	0.112
Cheek		5	1.6	11	3.5	16	2.6	0.121
Neck		5	1.6	0	0.0	5	0.8	0.026
Salivary gland—malignant		17	5.4	15	4.8	32	5.1	0.752
Salivary gland—benign		81	25.7	86	27.7	167	26.7	0.567
Thyroid		1	0.3	0	0.0	1	0.2	0.321
Lip		9	2.9	1	0.3	10	1.6	0.012
Maxillary sinus—malignant		9	2.9	8	2.6	17	2.7	0.832
Maxillary sinus—benign		11	3.5	8	2.6	19	3.0	0.507
CUP		0	0.0	3	1.0	3	0.5	0.080
Ear		0	0.0	1	0.3	1	0.2	0.313
Total		315	50.4	310	49.6	625	100.0	

**Table 2:** Cases of confirmed benign or malignant head and neck tumours before and during the COVID-19 pandemic based on the analysed variable.

	Period	Pre-COVID-19 Pandemic		COVID-19 Pandemic		Total		*p*-Value- Chi^2^ Test
Variable		*n*	%	*n*	%	*n*	%	
Diagnosis	malignant tumour	223	70.8	215	69.4	438	70.1	0.695
	benign tumour	92	29.2	95	30.6	187	29.9	
Cancer	primary	313	99.4	304	98.1	617	98.7	0.148
	recurrent	2	0.6	6	1.9	8	1.3	
Second cancer at another site	yes	12	3.8	14	4.5	26	4.2	0.658
	no	303	96.2	296	95.5	599	95.8	
Gender	female	115	36.5	127	41.0	242	38.7	0.252
	male	200	63.5	183	59.0	383	61.3	
Place of residence	urban area	197	62.5	245	79.0	442	70.7	<0.001
	rural area	118	37.5	65	21.0	183	29.3	
Planned treatment	radical	270	85.7	256	82.6	526	84.2	0.283
	palliative	45	14.3	54	17.4	99	15.8	
Admission	on the basis of a regular referral	262	83.2	237	76.5	499	79.8	0.036
	on the basis of a DILO card	53	16.8	73	23.5	126	20.2	

**Table 3:** Cases of confirmed malignant head and neck tumours before and during the COVID-19 pandemic based on the analysed variable.

	Period	Pre-COVID-19 Pandemic		COVID-19 Pandemic		Total		*p*-Value- Chi^2^ Test
Variable		*n*	%	*n*	%	*n*	%	
Gender	female	69	30.9	68	31.6	137	31.3	0.887
	male	154	69.1	147	68.4	301	68.7	
Place of residence	urban area	148	66.4	167	77.7	315	71.9	0.008
	rural area	75	33.6	48	22.3	123	28.1	
Planned treatment	radical	178	79.8	161	74.9	339	77.4	0.217
	palliative	45	20.2	54	25.1	99	22.6	
Admission	on the basis of a regular referral	184	82.5	154	71.6	338	77.2	0.007
	on the basis of a DILO card	39	17.5	61	28.4	100	22.8	

## Data Availability

The data are available from the authors of the manuscript after contacting the corresponding author.

## Abbreviations

HNC—head and neck cancer

DILO card—diagnosis and oncological treatment card

SARS-CoV-2—severe acute respiratory syndrome COVID-19

CUP—cancer of unknown primary

COVID-19—coronavirus disease 2019
